# Book Reviews and Notices

**Published:** 1888-11

**Authors:** 


### BOOK REVIEWS AND NOTICES.

INTRA-CRANIAL TUMORS. By BYROM BRAMWELL, M. D., F. R. C. P. E., F. R. S. E. Philadelphia: J. B. Lippincott Company. 1888.

The past year has been exceedingly rich in literature on intra-cranial surgery. The present volume is the most ambitious and satisfactory contribution from the clinical side. It is a model of the book-maker's and engraver's art. The photo-engravings of pathological specimens put the reader in the position of an original observer. This is not perhaps the least advantage it possesses over hand-engraving. It almost eliminates the personal equation, and renders the observations of to-day capable of interpretation from the standpoint of to-morrow. In all scientific works this form of engraving and illustration is to be encouraged.

It will be a disappointment to many readers that the author has excluded from consideration abscesses of the brain, although he includes other parasitic cysts to which they are etiologically and pathologically connected. Clinically, abscesses of the brain must always be excluded before a tumor (Bramwell) can be diagnosticated.

Among the symptoms of intra-cranial tumor, double-optic neuritis occupies a full share of attention. The author gives preference to the pressure-irritation theory of Leber and Deutschmann. It seems as if the subject of localization of the tumor had not received as full and careful consideration as its surgical importance requires. Recent clinical and pathological experience are not utilized to correct the well-known experimental conclusions of physiologists. In this respect the contributions of Horsley * and Seguin † are much more satisfactory. Arthur W. Hare adds a chapter on the surgical treatment of intra-cranial tumors.

A MANUAL OF GENERAL PATHOLOGY, Designed as an Introduction to the Practice of Medicine. By JOSEPH FRANK PAYNE, M. D., Oxon., F.R.C.P. Pp. xiv and 528. Philadelphia: Lea Brothers & Co. 1888. Chicago: A. C. McClurg & Co.

Those who are familiar with the development of pathological knowledge since the appearance of Virchow's *Cellular Pathologie*, have observed that the field of pathological histology has developed more rapidly than, and somewhat at the expense of, general pathology. The causes which have brought this state of things about are evident, and need not be mentioned in this connection. The American translation of Uhle and Wagner's General Pathology has, whatever the reasons may be, not come into general use among students; is, besides, now twelve years old, and consequently does not represent present opinions, especially in regard to parasites. In view of the reasons briefly outlined above, it should not seem an extravagant statement to say that this book, if a good one of its kind, is both a much-needed and a welcome contribution. It is an excellent book in every way. The author has succeeded very well in discussing his subject in its relation to the practice of medicine. It is a conspicuous fact that a book may be excellently arranged, thoroughly cover its subject, and fully represent the latest opinion, and yet fail to reach the readers for whom it is intended, through some fault in style and attractiveness. It is a pleasure to be able to say of the volume under consideration that it does not belong in that class among books. Its style is attractive and clear, and the comprehensiveness of the discussion of the various topics is admirable. The author is evidently a teacher of ability and long experience. If the book receives the attention among medical men which its merit demands, it will have a very large circle of careful readers.

An attempt to present an outline of the manner in which the subject is treated, is intentionally omitted. In the limits of a book-notice such an attempt is unavoidably abortive, in dealing with such a multitude of details as general pathology embraces. The work of the publishers leaves nothing to be desired. E. W.

A PRACTICAL TREATISE ON THE MEDICAL AND SURGICAL USES OF ELECTRICITY; including Localized and General Faradization; Localized and Central Galvanization; Franklinization; Electrolysis and Galvano-Cautery. By GEO. M. BEARD, A. M., M. D. and A. D. ROCKWELL, A. M., M. D. Sixth edition. Revised by A. D. ROCKWELL, M. D. New York: William Wood & Co. Chicago: W. T. Keener. Pp. xxx and 758. 1888.

Since the first appearance of this work it has been of recognized value—one of the best books in the English language—on the subject. Having now reached a sixth edition it is safe to say it is still regarded with as much favor, because of careful revision with each new edition. The changes that have been made in the different editions are so expressly stated in the preface to the sixth edition that, as the work is so well known, it only needs a

* Horsley; "Topography of the Cerebral Cortex." American Journal of the Medical Sciences, April, 1887, p. 342.

† Seguin: "Contribution to the Diagnosis and Surgical Treatment of Tumors of the Cerebrum," American Journal of Medical Sciences, August, 1888.

"Cerebral Localization." Annual of the Universal Medical Sciences for 1887.

statement of those changes, and that of the preface to this edition gives them so fully that we here produce it:

"The changes and additions in this work, as successive editions have been issued, have been confined to physics and physiology and the department of nervous diseases, where electricity has wrought its best results. While this agent is far from being a panacea, yet its increasing range of usefulness in medicine may be best illustrated by reference to these various additions. In the second edition the chapters on Electro-Physics and Physiology were largely rewritten; the method of central galvanization described and illustrated, electro-surgery more fully treated, and the relation of electricity to the diseases of children and of the skin considered in detail. In the third edition were given the highly satisfactory results following the treatment of exophthalmic goitre by galvanization of the sympathetic, and some of the sequelæ of acute diseases by general Faradization. The chapter on Electro-Diagnosis also was largely rewritten. A fourth edition was rendered necessary by a revival of the use of Franklinic electricity, due to vastly improved appliances, and contained also the extraordinary results following the application of dynamic electricity to cases of extra-uterine pregancy. The fifth edition discussed facts hitherto, and even now, but little appreciated, concerning the induction coil, its varieties, and the differential indications for their use. These statements the author deems worthy of careful consideration, and believes that further experience will result in still more important, as well as more definite, deductions. Within the past two or three years Apostoli, of Paris, has, by his experiments and the results that he has succeeded in obtaining, greatly enlarged the domain of electricity in gynæcology. The revision in the present edition, therefore, has been mainly restricted to this subject, and the chapter on the Diseases of Women almost entirely recast. The methods through which these better results in gynæcology are obtained do but confirm the truth of the observation made in the preface to the third edition, to the effect that the real scientific basis for the use of electricity in medicine and surgery is found in electro-physics more than in electro-physiology."

PHYSICIANS' INTERPRETER. IN FOUR LANGUAGES. Specially arranged for diagnosis. By M. VON V. Philadelphia: F. A. Davis. 1888.

The publisher announces that the object of this little work is to meet a need often keenly felt by the busy physician, namely: the need of some quick and reliable method of communicating intelligibly with patients of those nationalities and languages unfamiliar to the practitioner. The plan of the book is a systematic arrangement of questions upon the various branches of practical medicine, as the eye, ear, nose, throat, fevers, surgical operations, stomach complaints, general health, special diet, patient's history, etc., etc., and each question is so worded that the only answer required of the patient is merely YES or NO. The questions are all numbered, and a complete index renders them always available for quick reference, in English, French, German, and Italian.

It is bound in full Russia leather, for carrying in the pocket (size five by two and three-quarter inches, 206 pages).

The plan of the book is similar to that of the phrase-books, designed for the convenience of travelers in Continental Europe, who are not familiar with the languages of the different countries visited. This work will, no doubt, be found to serve a like purpose for some physicians, among whose patients several different languages are spoken.

HAND-BOOK FOR YOUNG AND OLD OPTICIANS. By W. BOHNE. With illustrations. Published by the author. New Orleans. 1888. Pp. 108.

This is a small guide-book, designed, as the author announces, for the guidance of opticians. He states that it was prepared for them because he was unable to find any similar book to aid him in his work, and he now gives them the result of his recorded experience as a practical optician. There is much in it of practical value to the working optician, and the advice given is generally reliable, even when he goes out of his province to advise treatment in cases of injury to eyes from various accidents, to "their friends and customers, who may, in their trouble, come running to the optician as the next proper person to give them relief."

If used as designed by the author, it will doubtless serve many well, but it should not be supposed that the instruction and advice given will qualify an optician to assume the function of the ophthalmologist, any more than reliable instruction for the pharmacist, as such, justifies him in assuming the prerogative of the physcian, a fact which too many disregard.

THERAPEUTICS; ITS PRINCIPLES AND PRACTICE. By H. C. WOOD, M. D., LL. D., Professor of Materia Medica and Therapeutics, and Clinical Professor of Diseases of the Nervous System in the University of Pennsylvania. A Treatise on Therapeutics. Seventh edition. Rearranged, rewritten, and enlarged. Philadelphia: J. B. Lippincott Company. Pp. 908. 1888.

That the seventh edition of this work, so familiar to the profession, has already been issued, is conclusive of the estimate in which it is held. No more is, therefore, required to be said regarding it than to notice some of the points in which this edition differs from the preceding six.

The author justly says: "Comparatively few persons have a full conception of the rapid progress of therapeutics, and the amount of labor involved in keeping up with this forward movement."

Nearly two hundred pages of new matter have been added to the book.

All the new drugs of any recognized merit have been considered, and in the therapeutic portion, the remedial measures that have been employed by the profession in recent years have been here presented. The book can safely be accepted as an exponent of the deserving materia medica and the enlightened therapeutics of the present day.

The mechanical work of the book is excellent. It is printed on good paper with clear type, and is in every way worthy of the publishing house of J. B. Lippincott Company.

TRANSACTIONS OF THE ASSOCIATION OF AMERICAN PHYSICIANS. Second session. 1887. Pp. xx and 254.

The report of the transactions of the association gives the seventeen papers read before it at its second annual meeting, together with the discussion of them.

A list of the one hundred members, to which the association limits its membership, and of the honorary members, is given, as well as a brief biographical sketch of each of the three members, Drs. Hudson, McBride, and Rochester, who died during the year.

A PRACTICAL TREATISE ON DISEASES OF THE EYE. By EDOUARD MEYER, Professor A L'École Pratique de Mèdicin de Paris, Chevalier of the Legion of Honor," etc., etc. Translated by FREELAND FERGUS, M.D. With two hundred and sixty-seven illustrations and three colored plates. Philadelphia: P. Blakiston, Son & Co. Pp. xi and 647. 1887.

This is the first Engl.sh edition of the work of the author, published in French in 1872, and, since then, translated into the German, Spanish, Polish, Russian, and Japanese languages. The present translation is from the third French edition.

The translator says: "The original work struck me as being not only the most concise, but, also, the most comprehensive manual on the branch of which it treats that I had ever perused, and it was this conviction which led me to undertake the translation."

The fact of the work having been translated into so many languages, with four editions of the German translation, indicates that the English translator's conviction regarding the merits of the book is shared by many others. The chromo-lithographic plates are from the well-known and justly esteemed atlas of Dr. Liebreich. The text of the work is systematically arranged, being divided into twelve chapters. It is abundantly illustrated with wood engravings of a high order of merit.

The American publishers have issued it in their accustomed good form, so well known to the medical profession.

It is a pleasure to commend a book so worthy of commendation as this one from every point of view. It merits a large circulation among English-speaking ophthalmologists.

NASAL POLYPUS, WITH NEURALGIA, HAY FEVER, AND ASTHMA, IN RELATION TO ETHMOIDITIS. By EDWARD WOAKES, M. D., London, Senior Aural Surgeon and Lecturer on Diseases of the Ear at the London Hospital; Surgeon to the London Throat Hospital. With illustrations. Philadelphia: P. Blakiston, Son & Co. Pp. vii and 140. 1887.

The chief object of this little book, it is stated by the author, is to discuss the subject of ethmoiditis, and to present its claims upon the profession for more consideration than it has usually received. Other affections of that region are incidentally considered, whether standing in the relation of cause or consequence, or as a complication of that affection. The book is divided into twelve chapters, and treats of a subject that is now attracting much attention, for it is not yet clearly established as to just what relation, from an etiological standpoint, affections of the naso-pharynx hold to affections of the ear. There is concurrence of opinion that there is an intimate relation between them, and in the effort that is being made to get facts and formulate ideas regarding the pathology, this book is timely, and will be found to be useful. The merit of the previous writings of the same author on kindred subjects has been recognized, and this one is welcomed as bringing further contributions where and when wanted. The cuts given are well executed, and aid in elucidating the text instead of confusing, as not infrequently happens.

CHEMICAL EXPERIMENTS FOR MEDICAL STUDENTS; Arranged after Beilstein. By W. S. CHRISTOPHER, M. D. Cincinnati: Robert Clarke & Co. Pp. 84. 1888.

The author says in his preface that "the object has been to simplify and limit, rather than to multiply, the number of experiments," and "the application of the experiments in inorganic chemistry to systematic qualitative analysis has been omitted, as not adapted to the wants of the medical student."

In the limited time available for practical chemistry in most medical colleges, it becomes desirable for the student to acquire the general principles of the science, and such special facts as may be most useful to him in practice, as directly as possible; to study chemistry from the medical rather than from the chemical standpoint.

The experiments cover work with the principal metals and acids, using Beilstein's examples. In addition, the more important alkaloids and some organic compounds of medical interest are considered. In physiological chemistry, the work deals with the proteids and carbo-hydrates, the digestive processes, blood, bile, milk, and urine.

The acid tests recently introduced for the clinical examination of stomach contents are also given.

It may be used in conjunction with any systematic treatise on the subject.

Extra blank leaves are inserted at the end for students' notes.

A SYNOPSIS OF THE MEDICAL BOTANY OF THE UNITED STATES. By J. M. G. CARTER, M. A., M. D., Ph.D., Sc.D., Member of the American Medical Association. Pp. 176. St. Louis. G. H. Field. 1888.

In his introduction the author gives the number of the medical plants in the United States as embracing "about 140 orders, 620 genera, and more than 1,300 species and varieties." The statement is also made that "the list is fairly complete of plants east of the Mississippi River," and that, regarding those in the vast country lying west of that river, "we must feel that our medical knowledge of that region is comparatively meagre."

The parts of the plants from which their active principles are taken, and to which their remedial virtues are due, are given, and attention is drawn to the established fact of the changes which result in plants cultivated elsewhere than in their native soil, citing the fact that cinchona plants, growing in hot-houses, are nearly free from quinine, etc., etc. The work indicates care and labor in its preparation, and contains interesting and useful information.

A CLINICAL ATLAS OF VENEREAL AND SKIN DISEASES, INCLUDING DIAGNOSIS, PROGNOSIS AND TREATMENT. By ROBERT W. TAYLOR, A. M., M. D. Parts I and II. Venereal Diseases. Philadelphia: Lea Brothers & Company. 1888.

If the demand for this atlas is commensurate with the need for it among the profession it will be large indeed. It will be of no small value even to specialists, and to those physicians educated at the many medical colleges of this country which are so conspicuously lacking in both clinical material and teaching, it will prove a veritable boon. The text is admirable in every way, and the presswork and general appearance are excellent. Of course, in all books of this class the carping critic finds fault with the colors in the lithographic plates. This inability to accurately reproduce the natural colors seems liable to prove a lasting stumbling-block to the lithographer's art. In this particular atlas, however, the coloring is fully up to the average, and the selection of subjects has been made with admirable judgment, the aim of the editor being to present clear types rather than exceptional cases.

The work is to be completed in eight folio parts, measuring 14 x 18 inches, embracing fifty-eight colored plates, numerous figures and engravings, and about 400 pages of text.

MANUAL FOR HOSPITAL NURSES, AND OTHERS ENGAGED IN CARING FOR THE SICK. By EDWARD J. DOMVILLE, L. R. C. P. Lond., etc., etc. With Recipes for Sick-Room Cookery. Sixth edition. Philadelphia: P. Blakiston, Son & Co. Chicago: W. T. Keener. 1888. Pp. iv and 100.

This manual contains much information of immediate use to those engaged in the care of sick, and will be found a great convenience to the pupil-nurses in the various training-schools for nurses. It takes a comprehensive view of the subject, and presents the dignity, as well as the responsibility, of the position of a nurse. In civilized communities persons are no longer engaged as "nurses" because they are too ignorant, or lazy, or indifferent to do anything else than engage in so-called nursing. It has now become a profession—as useful, and, in many respects, as honorable, as the medical profession, and only by the proper co-operation of the two can the best results be obtained.

The instructions given in the manual cover the whole field of the duties of the faithful nurse, and cannot fail to benefit all who will study them intelligently. The work can be commended, unqualifiedly.

ANÆSTHETICS; THEIR USES AND ADMINISTRATION. By DUDLEY WILMOT BUXTON, M. D., B. S., Member of the Royal College of Physicians, etc., etc. Philadelphia: P. Blakiston, Son & Co. Chicago: W. T. Keener. Pp. xii and 164. 1888.

One aim of this little manual is to direct attention to the fact that anæsthetics are administered too often by inexperienced and incompetent persons—those who do not realize the responsibility which attaches to their administration.

The work is divided into twelve chapters, the first of them being devoted to the history of anæsthesia and anæsthetics. Then the subject of the preparation of the patient for an anæsthetic, and the choice of the one to be used, bearing in mind the age and condition of the patient, pathological condition, idiosyncrasy, etc., etc., the different general anæsthetics, and their combination. Inhalers, accidents and their treatment, local anæsthetics, the medico-legal aspects of the administration of anæsthetics, etc., etc., are considered. The work presents the subject from a very practical standpoint. It contains much useful information, which is presented in a condensed form. It is interesting reading, and observance of the suggestions made will do much to guard against avoidable accidents, which occur too frequently.

INDEX CATALOGUE OF THE LIBRARY OF THE SURGEON-GENERAL'S OFFICE, U. S. ARMY. Volume IX.

The ninth volume of the valuable index catalogue has just been issued, and begins with "Medicine (Popular)" and ends with "Nywelt." It has been prepared with the same care, and issued in the same form as its predecessors, that are so well known to the profession, and which are so creditable to the medical department of the army, and to the Government whose liberality provides for the issue of the catalogue.

THEINE IN THE TREATMENT OF NEURALGIA. Being a Physiological Contribution to the Therapeutics of Pain. By THOMAS J. MAYS, M. D. Philadelphia: P. Blakiston, Son & Co. Chicago: W. T. Keener. Pp. 84. 1888.

This is a reprint from the *Polyclinic*, and is issued in the form of a small manual, consisting of four chapters, an appendix, and an index.

The first chapter gives a history of the tea-plant, and the discovery of the alkaloid theine (in 1827) found therein, as well as a description of the alkaloid.

The second chapter describes the physiological action of theine.

The third chapter gives the special therapeutic indications for the use of theine.

The fourth chapter is devoted to the consideration of forty-eight cases of neuralgia, in its varying forms, in which theine was used, and presumably with marked benefit. It might not have been amiss to have added another chapter and given the other side of the picture—the evil results of excessive use of infusion of the tea-plant, including this alkaloid.

PTOMAINES AND LEUCOMAINES, OR THE PUTREFACTIVE AND PHYSIOLOGICAL ALKALOIDS. By VICTOR C. VAUGHAN, Ph. D., M. D., and FREDERICK G. NOVY, M. S. Philadelphia: Lea Brothers & Co. Chicago: A. C. McClurg & Co.

This is a book which deserves special attention. It is the first attempt which has been made to present in book form the literature of the ptomaines and leucomaines, and the authors have succeeded in putting in succinct form the pith of the various contributions on the subjects. The citation of the contributions is very valuable, and covers some twenty pages.

The book makes it evident that those who make any pretensions to the application of the general principles of chemistry, bacteriology, or physiology to the practice of medicine cannot afford to be ignorant of the general principles involved in the study of the ptomaines and leucomaines.

The following constitute the chapter headings: Definition and Historical Sketch of Ptomaines, The Relation of Ptomaines to Disease, The Importance of Ptomaines to the Toxicologist, Methods of Extracting Ptomaines, Chemistry of the Ptomaines, Chemistry of the Leucomaines, The Pathological Importance of the Leucomaines, Literature.

The authors are to be complimented for presenting to the profession a compendium of valuable information. Pp. 314. 1888.

MANUAL OF CHEMISTRY. By W. SIMON, Ph. D., M. D. Second edition. Thoroughly revised and greatly enlarged. Philadelphia: Lea Brothers & Co. Chicago: A. C. McClurg & Co. 1888.

This is a regular text-book on chemistry, intended for students in pharmacy and medicine. It is illustrated by colored plates to make more intelligible the various reactions in which color forms an important item. A series of questions are found at intervals through the book, so that the student may ascertain for himself how far he has appropriated the information imparted. The text is clear, and the general presentation of the book, as a whole, will compare well with other recent text-books on the same subject.

A TEXT-BOOK OF PHARMACOLOGY, THERAPEUTICS, AND MATERIA MEDICA. By T. LANDER BRUNTON, M. D., D. Sc., F. R. S. Adapted to the U. S. P. by FRANCIS H. WILLIAMS, M. D. Third edition. Philadelphia: Lea Bros. & Co. Chicago: A. C. McClurg & Co.

The general title, and the reputation of the author, will sufficiently indicate to the student the character of the book. As will be seen, it is the third edition, and the author has, as far as possible, made it conform to the ideas of modern investigation. The struggle for existence between the organism and the microbes which invade it, as presented by Metschnikoff is well illustrated. The absorption and elimination of ptomaines and leucomaines receive attention; in fact, the whole chapter on the action of drugs on protoplasm, blood, and low organisms, some fifty pages, will be found as complete as is practicable under the circumstances. The book is well printed, and creditable in every way. It will be found to be a convenient book for reference by physicians and pharmacists.

